# Supplementary material for: Effect of AOX1 and GAP transcriptional terminators on transcript levels of both the heterologous and the GAPDH genes and the extracellular Yp/x in GAP promoter-based Komagataella phaffii strains
Source: PeerJ. 2024 Sep 26;12:e18181. doi: 10.7717/peerj.18181 (PMC11922483; doi:10.7717/peerj.18181)
Supplement: Supplemental Information 1 [file peerj-12-18181-s001.pdf]

Table S1 Oligonucleotides (primers) and probes.

Primer	Sequence
T17AP	GACTCGAGTCGACATCGAT$_{17}$
RACEAP	GACTCGAGTCGACATCG
5qGAP	GGAGACTACCTACGAGGAGATCAA
FTE1	CGCTGACAAGAACGGTTACA
FTE2	CTTGTGGGTGGAAACCAA
3TH	GGTGCCGAGGATGACGATGA
GAPF	GTCCCTATTTCAATCAATTGAA
5ACT	GCCCCAGAAGAGCACCCAGT
3ACT	ACCGGAAGCGTACAGGGACA
5PTFTEII	TCCAAATCACTGACGGTCCAGA
3PTFTEII	TCACCGTCTTGAGCAACGAACA
5PTGAPDH	TTCCATCTTCCACTGGTGCT
3PTGAPDH	TTGGGACACGGAAAGCCAAA
5qYPT1	GCGGTCAAGCTAGTGCTTCT
3qYPT1	TGCTGACAAAACGTAAAACCA

Probe	Sequence
pFTEII	TTTGGGTTTCGGTTTGGGTCCAGA
pGAPDH	CCAGAATTGAACGGTAAGCTGA
