# Supplementary figures and images for: Effect of AOX1 and GAP transcriptional terminators on transcript levels of both the heterologous and the GAPDH genes and the extracellular Yp/x in GAP promoter-based Komagataella phaffii strains
Source: PeerJ. 2024 Sep 26;12:e18181. doi: 10.7717/peerj.18181 (PMC11922483; doi:10.7717/peerj.18181)

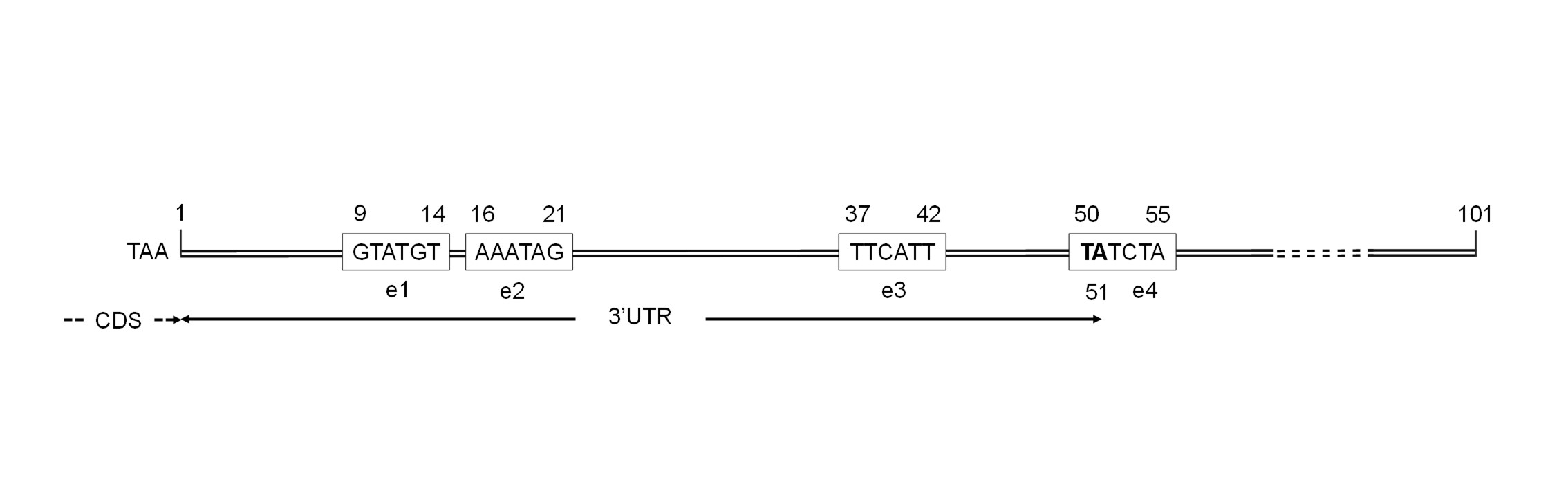

Supplement: Supplemental Information 2 — The 3′-untranslated region (3′UTR) is indicated with a double-headed arrow. The numbers correspond to nucleotide positions from the 3′UTR start site. The putative 3′-processing elements e1, e2, e3, and e4, also known as efficiency, positioning, near-upstream, and near-downstream elements, respectively, are shown in boxes. The stop codon (TAA) of the GAPDH coding sequence (CDS) and the TA sequence (in bold) for the putative RNA cleavage and polyadenylation site are also shown. [file peerj-12-18181-s002.jpg]

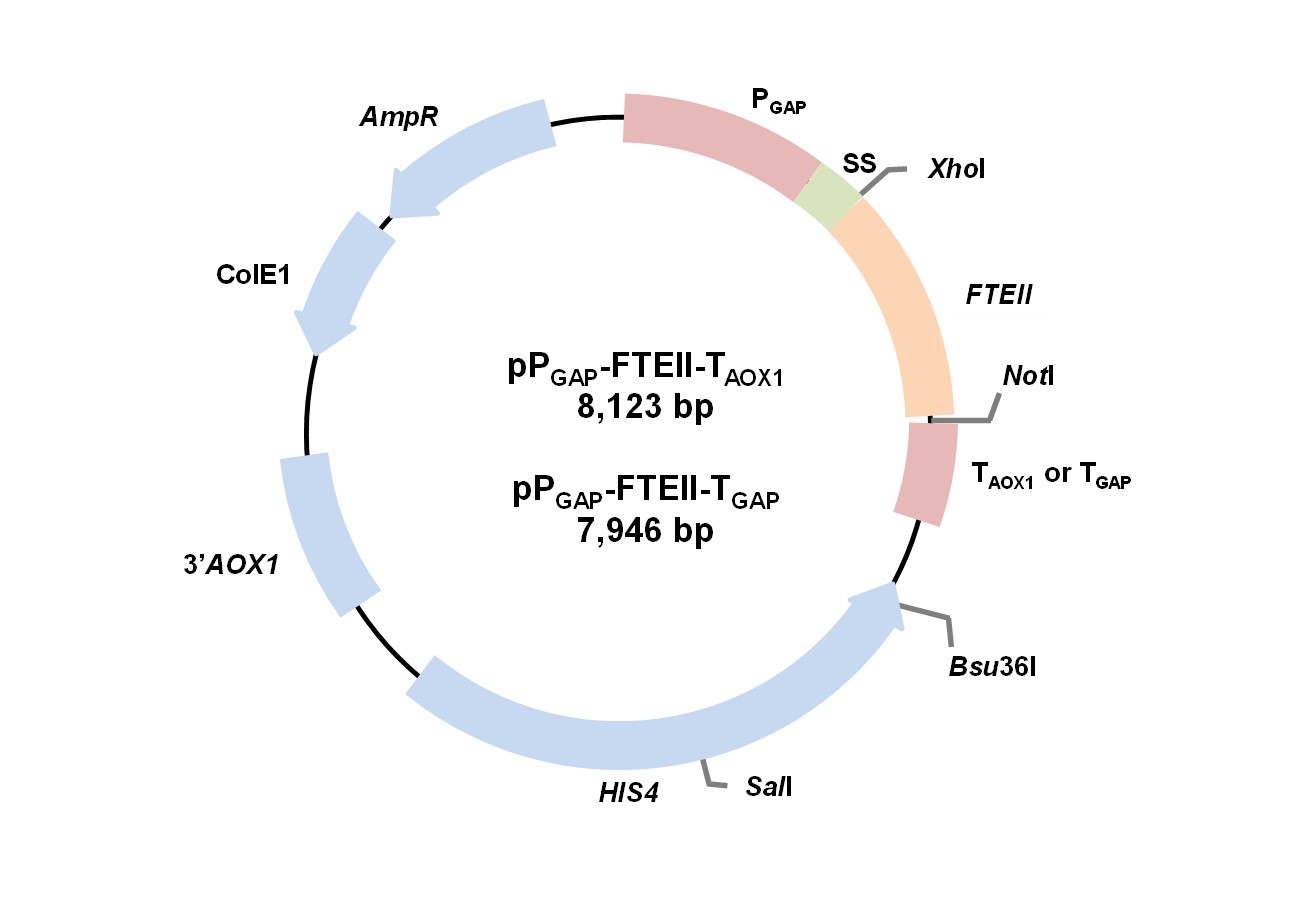

Supplement: Supplemental Information 3 — PGAP, promoter of the GAPDH gene; SS, alpha-factor prepro-secretion signal coding sequence; FTEII, gene coding for the mature beta-propeller phytase FTEII; TAOX1, AOX1 transcriptional terminator; TGAP, transcriptional terminator of the GAPDH gene; HIS4, K. phaffii wild-type gene coding for histidinol dehydrogenase; 3’AOX1, AOX1 downstream region; ColE1, Escherichia coli origin of replication; AmpR, ampicillin resistance gene; NotI and Bsu36I, restriction sites for cloning the TGAP sequence; SalI, restriction site for vector linearization before K. phaffii transformation by electroporation. [file peerj-12-18181-s003.jpg]

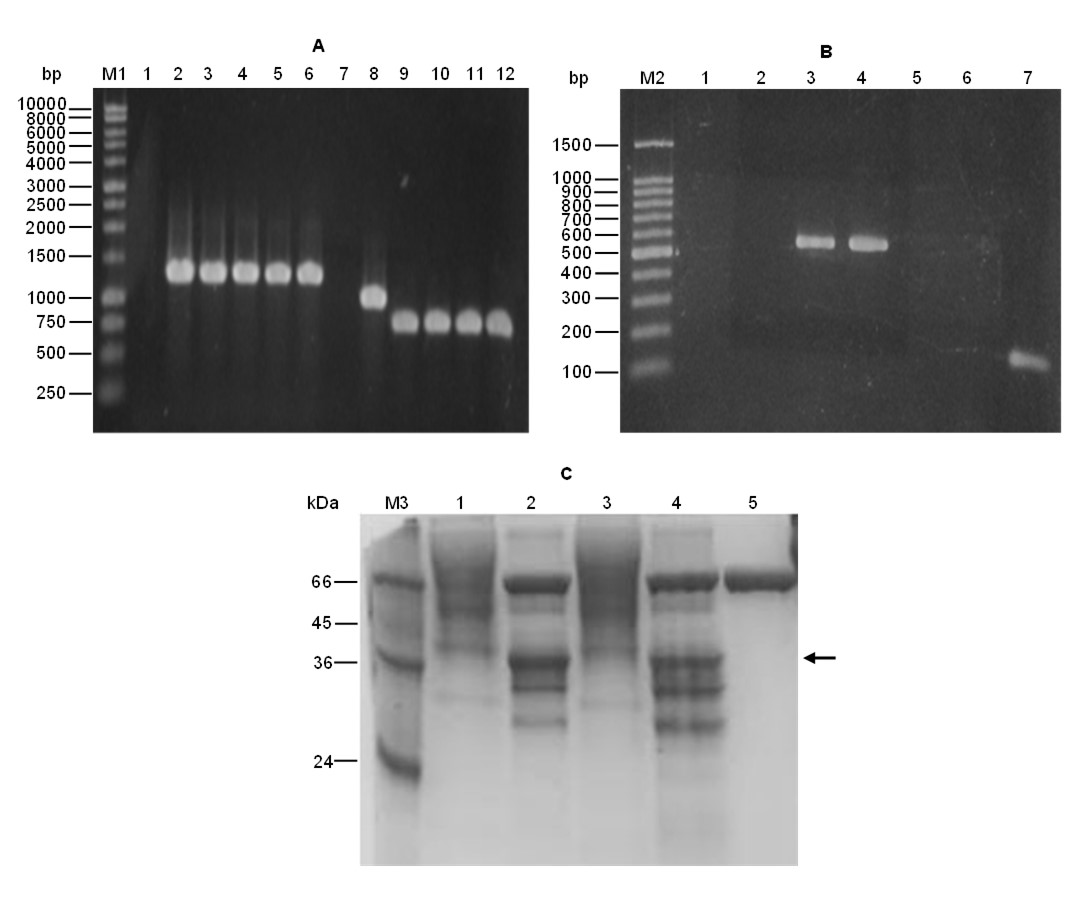

Supplement: Supplemental Information 4 — (A) Lane M1, molecular size marker; lanes 1 and 7, negative controls of PCR; lane 2, PCR product of genomic DNA from the PGAP-TAOX1-based strain with the 5’GAPF/FTE2 primer pair; lanes 3 to 6, PCR product of genomic DNA from four clones of the PGAP-TGAP-based strain with the 5’GAPF/FTE2 primer pair; lane 8, PCR product of genomic DNA from the PGAP-TAOX1-based strain with the FTE1/3TH primer pair; lanes 9 to 12, PCR product of genomic DNA from four clones of the PGAP-TGAP-based strain with the FTE1/3TH primer pair. (B) Lane M2, molecular size marker; lanes 1 to 4, assays with the FTE1 and FTE2 primers; lane 1, negative control of the reverse transcriptase step; lanes 2, PCR negative control; lanes 3, RT-PCR product from the PGAP-TAOX1-based strain; lane 4, RT-PCR product from the PGAP-TGAP-based strain; lanes 5 to 7, assays with the actin (5ACT and 3ACT) primers; lane 5, negative control of the reverse transcriptase step; lane 6, PCR negative control; lane 7, RT-PCR amplified product (positive control). (C) Lane M3, molecular mass marker; lanes 1 and 2, proteins without and with Endo Hf glycosidase from the PGAP-TAOX1-based strain; lanes 3 and 4, proteins without and with Endo Hf glycosidase from the PGAP-TGAP-based strain; lane 5, Endo Hf glycosidase. The arrow indicates N-deglycosylated recombinant phytase FTEII. [file peerj-12-18181-s004.jpg]
